# Evaluation of Oromucosal Natural Gum-Based Emulgels as Novel Strategy for Photodynamic Therapy of Oral Premalignant Lesions

**DOI:** 10.3390/pharmaceutics15102512

**Published:** 2023-10-23

**Authors:** Emilia Szymańska, Joanna Potaś, Marcin Baranowski, Robert Czarnomysy, Magdalena Ewa Sulewska, Anna Basa, Małgorzata Pietruska, Krzysztof Bielawski, Katarzyna Winnicka

**Affiliations:** 1 Department of Pharmaceutical Technology, Medical University of Białystok, Mickiewicza 2c, 15-222 Białystok, Poland; joanna.potas@umb.edu.pl (J.P.); katarzyna.winnicka@umb.edu.pl (K.W.); 2 Department of Physiology, Medical University of Bialystok, Mickiewicza 2c, 15-222 Białystok, Poland; marcin.baranowski@umb.edu.pl; 3 Department of Synthesis and Technology of Drugs, Medical University of Białystok, Jana Kilińskiego 1, 15-089 Bialystok, Poland; robert.czarnomysy@umb.edu.pl (R.C.); krzysztof.bielawski@umb.edu.pl (K.B.); 4 Department of Periodontal and Oral Mucosa Diseases, Medical University of Białystok, ul. Waszyngtona 13, 15-269 Białystok, Poland; magdalena.sulewska@umb.edu.pl (M.E.S.); mpietruska@wp.pl (M.P.); 5 Faculty of Chemistry, University of Bialystok, Ciołkowskiego 1K, 15-245 Białystok, Poland; abasa@uwb.edu.pl

**Keywords:** photodynamic therapy, delta-aminolevulinic acid, oromucosal carrier, emulgel, tragacanth, xanthan, gellan gum

## Abstract

Photodynamic therapy (PDT) recently has been shown as a promising option in the treatment of premalignant lesions of the soft oral tissues. Effective delivery of photosensitizer is challenging due to poor drug adherence to the oromucosal epithelium. In the present work, emulgels composed of natural polysaccharide gums (tragacanth, xanthan and gellan) were evaluated as novel oromucosal platforms of delta-aminolevulinic acid (ALA) for PDT. Apart from mucoadhesive and textural analysis, the specific steps involved studies on drug penetration behavior and safety profile using a three-dimensional human oral epithelium model (HOE). All designed emulgels presented greater mucoadhesiveness when compared to commercial oromucosal gel. Incorporation of ALA affected textural properties of emulgels, and tragacanth/xanthan formulation with greater hardness and cohesiveness exhibited a protective function against the mechanical tongue stress. Permeability studies revealed that ALA is capable of penetrating across oromucosal epithelium by passive transport and all formulations promoted its absorption rate when compared to a commercial topical product with ALA. Importantly, the combination of tragacanth and xanthan profoundly enhanced photosensitizer retention in the buccal epithelium. Tested samples performed negligible reduction in cell viability and moderately low IL-1β release, confirming their non-irritancy and compatibility with HOE. Overall, the presented findings indicate that tragacanth/xanthan emulgel holds promise as an oromucosal ALA-carrier for PDT strategy.

## 1. Introduction

Photodynamic therapy (PDT) is a noninvasive therapeutic option for the management of inflammatory skin and mucosal diseases. The fundamental elements of PDT include a photosensitizer, a specific wavelength of visible light and oxygen. Specifically, a photosensitizer is administered in a form of prodrug and accumulates in abnormal cells. Upon exposure to light, it undergoes transition to its active state and reacts with endogenous oxygen, contributing to the formation of reactive oxygen species. This causes an irreversible damage of targeted cells by a complex cascade of biological, chemical and physiological reactions [1,2].

Although a variety of photosensitizers have been tested for PDT [3,4,5], 5-aminolevulinic acid hydrochloride (ALA) is considered one of the most effective agents due to high selectivity and a short half-life [6]. ALA, belonging to the class of alpha amino ketones, is a naturally occurring precursor involved in the biosynthetic pathway of porphyrins, in particular protoporphyrin IX (PpIX). After administration, ALA penetrates into cells, where it is metabolized to an active PpIX. Subsequent activation by infrared light with a wavelength range of 600–800 nm leads to the formation of highly reactive singlet oxygen, which is responsible for cell destruction. ALA-PDT treatment is considered as a selective, well tolerated and minimally invasive therapeutic option with low risk of either allergic contact dermatitis or systemic side effects [6,7]. The accumulation of PpIX is much more pronounced in abnormal cells than in normal cells and hence there is rare risk of damage of underlying functional tissues. Topical ALA-PDT has been successfully applied for the treatment of malignant lesions, e.g., basal cell carcinoma, actinic keratosis, Bowen’s disease, vulval epithelial neoplasia and vulval Paget’s disease [2,6]. Recently, ALA-PDT has become an attractive alternative in the treatment of oral premalignant diseases, including oral lichen planus, erythroplakia and leukoplakia [8,9,10]. It is considered an efficacious therapeutic option to treat oromucosal lesions unresponsive to corticosteroids. Additionally, in contrast to standard surgical procedures or corticosteroids treatment, ALA-PDT displays a lower rate of disease reoccurrence [9].

A major challenge in effective oromucosal drug delivery is to assure its prolonged retention in the oral mucosa. In addition, ALA penetration is considered limited [11], requiring a proper time of retention on the tissue before exposure to light. Therefore, mucoadhesive drug carriers providing intimate and prolonged contact between drug and oral mucosa represent an attractive and noninvasive approach to improve local drug availability [12].

Mucoadhesion is referred to as the interfacial interaction between material and mucosal membrane which enables material to join with tissue and be retained on its surface. The mode of mucoadhesion comprises a contact stage with a subsequent consolidation stage. The latter involves complex physicochemical interactions including ionic, covalent, hydrogen or electrostatic forces [13]. Among a variety of mucoadhesive agents, natural gums, including tragacanth, xanthan and gellan, have gained particular interest in development studies on mucosal drug delivery systems, including buccal, vaginal, nasal and oral [14,15,16,17]. Tragacanth (TG) is a natural gum derived from *Astragalus* sp. Chemically, it is composed of two fractions: water soluble tragacanthin (30–40%) and water-swellable bassorin (or tragacanthic acid, 60–70%). The first one is a highly branched fraction with different monosaccharides including l-arabinose, l-fucose, d-mannose and d-glucose, whereas the latter is composed mainly of l-fructose, d-galacturonic acid, d-galactose, l-rhamnose and d-xylose [15]. Xanthan gum (XA) is a high-molecular-weight polysaccharide obtained from *Xanthomonas campestris* composed of a β (1–4)-d-glucopyranose glucan backbone with side chains of (1–3)-α-d-mannopyranose-(2–1)-β-d-glucuronic acid-(4–1)-β-d-mannopyranose [18]. TG and XA dissolve freely in water, forming transparent, viscous dispersions. Both are considered as stable and biocompatible in the generally regarded as safe (GRAS) category [19]. Gellan gum (GG) is a water-soluble negatively charged polysaccharide produced in a fermentation process by bacterium *Sphingomonas elodea*. It consists of the tetrasaccharide units composed of D—galactose, L—rhamnose and D—glucuronic acid. There are two different types of gellan gum: low and high acyl. The high-acyl GG gelifies upon cooling (at a temperature of about 65 °C) forming milky, elastic gels, while the low-acyl type creates firm, transparent, non-elastic gels in the presence of cations (mainly calcium ions) [20]. Due to emulsifying and viscosity enhancement properties, TG, XA and GG are used in food and cosmetics as rheology modifiers, suspending agents and stabilizers. In the pharmaceutical technology, these gums are also applied as tablet binders, or mucoadhesive agents for solid or semisolid dosage forms [18]. 

This study aimed to evaluate emulgels composed of naturally derived polysaccharide gums TG, XA and GG as oromucosal delivery platforms containing ALA for PDT. We hypothesized that the designed formulations facilitate drug presence at the mucosal site and serve as coating carriers which protect the damaged epithelial layers. The precise effort was made to distinguish differences in mucoadhesive, dissolution and ALA-penetration behavior between designed formulations. Additionally, broad in vitro studies on the safety profile, including irritation tests, a cytokine release assay and histopathological analysis in human three-dimensional oral mucosa tissue were performed. 

## 2. Materials and Methods

### 2.1. Materials

Tragacanth from *Astragalus gummifer*, composed primarily of tragacanthin and bassorin with average viscosity of 1% aqueous dispersion 200 cPa·s at 25 °C and XA (average viscosity of 1% aqueous dispersion 1500 cPa·s at 25 °C) were purchased from Sigma-Aldrich (St. Louis, MO, USA). Gellan gum (Kelcogel CG-HA) was obtained from CP Kelco (Atlanta, GA, USA). The simulated saliva fluid (SSF), composed of 0.1 M disodium hydrogen phosphate and 0.1 M potassium dihydrogen phosphate, was prepared according to [21]. Commercial topical gel with ALA Ameluz (serial number 04150094218327) composed of ALA, disodium phosphate isopropyl alcohol, polysorbate 80, propylene glycol, water, sodium benzoate, sodium dihydrogen phosphate dihydrate, xanthan gum, soybean phosphatidylcholine and medium chains triglyceride was purchased from Biofronterra Pharma GmbH (Leverkusen, Germany). Reference oromucosal gel Anaftin (serial number 270141) composed of water, polyvinylpyrrolidone, maltodextrin, propylene glycol, PEG-40 hydrogenated castor oil, xanthan gum, potassium sorbate, sodium benzoate, sodium hyaluronate, benzalkonium chloride, disodium dihydrogen ethylenediaminetetraacetate, sodium saccharin, dipotassium glycyrrhizinate and Aloe barbadensis was from Alliance Pharma Srl (Milan, Italy). All the other chemicals used in the studies are summarized in Table 1. Freshly excised porcine buccal mucosa was obtained from the veterinary service of a local slaughterhouse (Turośń Kościelna, Poland). Tissue specimens were preserved in the isotonic saline solution, frozen at −20 °C directly after killing the animal and kept no longer than 30 days. Prior to the test, tissue was thawed at ambient conditions, cut into pieces, and microscopically checked for tissue integrity.

### 2.2. Emulgel Preparation

Emulgels were prepared by the homogenization technique. During the formulation stage, the goal was to select an optimal polymers ratio and adjust polymer concentration in order to obtain comparable viscosities between designed formulations and control oromucosal gel Anaftin. Briefly, TG in combination with XA (formulation F1) or TG solely (formulation F2) was gradually dispersed in water (in a ratio 6.0/86.3 for F1 and 5.0/87.3 for F2, respectively) and homogenized in an automatic mixing system (1400 rpm for 25 min, Unguator E/S Eprus, Poland). For formulation F3, GG was carefully dispersed in water at temperature 80 °C (polymer to water ratio 0.7/89.6), cooled to 30 °C and homogenized with TG in an automatic homogenizing system (1400 rpm, 25 min). Subsequently, a water solution of preservatives and lecithin in propylene glycol was successively added to gel bases under constant stirring. After that, the ricin oil was emulsified to base. ALA was dispersed in propylene glycol and homogenized with emulgel bases with the final concentration of 5% (*w/w*). Emulgels composition is shown in Table 2. All formulations were kept in closed containers at 4 °C. Drug-free emulgel bases B1–B3 were additionally prepared for textural, mucoadhesive and safety studies. The pH of each sample was measured in three repetitions by a glass electrode of pH-meter Orion 3 Star (Thermo Scientific, Waltham, MA, USA).

### 2.3. SEM Analysis

Scanning electron microscopy (SEM) analysis was performed to evaluate the emulgels’ morphology. Prior to analysis, samples were placed into Petri dishes, frozen at −80 °C and dehydrated using freeze-drying (Alpha 2-4 LSC basic, Martin Christ, Germany) for 24 h at 10 mBa. Next, the samples were sputter-coated with gold (about 6.0 nm) in an argon atmosphere (Leica EM AC 2000, Wetzlar, Germany) and imaged using a scanning electron microscope (Inspect S50, FEI Company, Hillsboro, OR, USA). Duplicate analysis was performed for each sample.

### 2.4. Fourier Transform *Infrared*—*Attenuated Total Reflectance Spectroscopy* (ATR-FTIR)

ATR-FTIR analysis was applied to identify plausible interactions between ALA and polymers within emulgel composition. Infrared spectra were recorded in duplicate by the attenuated total reflectance method using a Nicolet 6700 ATR-FTIR spectrometer (Thermo Scientific, Dreieich, Germany) equipped with a DLaTGS detector and diamond ATR Smart Orbit accessory in the 4000 and 600 cm^−1^ range. Due to the short time of analysis, the moisture of all samples remained unaffected during spectral acquisition. 

### 2.5. HPLC Analysis

ALA content and drug distribution uniformity within emulgels were evaluated using a reverse-phase high-pressure liquid chromatography system (ProStar, Varian Inc., Palo Alto, CA, USA) equipped with a quaternary pump (Model 240), autosampler (Model 410), and fluorescence detector (Model 363) according to [22]. For this purpose, the drug was extracted from dosage form using the freeze—thaw technique in a mixture of PBS and methanol (4:1, *v/v*). Each sample was vigorously shaken for 5 min at 30 °C and then frozen for 15 min at −20 °C. The procedure was repeated three times. Separation was achieved on an Agilent OmniSpher 5 C18, 5 μm, 4.6 × 150 mm column (Santa Clara, CA, USA) following fluorescence derivatization of ALA. Briefly, 100 μL of sample was reacted with 100 μL of 0.1% (*w/v*) fluorescamine solution in acetonitryl and 300 μL of 0.1 M borate buffer (pH 8.0) for 10 min at ambient conditions. The mobile phase was acetonitrile/water (3:7, *v*/*v*) containing 0.1% trifluoroacetic acid. The flow rate was 1 mL/min, column temperature was maintained at 30 °C, and the excitation and emission wavelengths were 395 and 480 nm, respectively. The calibration curve was linear in the range from 37.5 to 25000 ng/mL (R^2^ = 0.999), with the lowest limit of detection of 6 ng/mL. The accuracy was 99.0 and 97.3% for ALA concentrations of 5000 and 150 ng/mL, respectively. The intraday precision (%CV) for these concentrations was 0.6 and 2.4%, respectively.

### 2.6. Textural Properties

Texture analysis was conducted by backwards extrusion method using a TA.XT Plus Texture Analyser (Stable Micro Systems; Godalming, UK). Each formulation (30.0 ± 0.1 g) was compressed with a disc (35 mm) with a speed of 2 mm/s into the sample to a defined depth of 10 mm at 25 ± 2 °C. Textural characteristics were determined as hardness (maximal force attained during the compression of emulgel), consistency (work required to deform the sample upon compression) and cohesiveness (force assessed upon upward movement of the disc). During measurements, the graph (force versus time) was plotted and textural properties were calculated using Texture Exponent 32 software. The results are shown as the average of three independent experiments.

### 2.7. Ex Vivo Mucoadhesive Studies

Mucoadhesive behavior of emulgels in contact with excised porcine buccal mucosa was performed with a texture analyzer, the TA.XT. Plus (Stable Microsystems, Godalming, UK). Drug-free or drug-loaded formulation samples (1.0 mL) were set on the upper G/Muc probe and secured with the attached support collar to keep the sample still while it was setting. The tissue was fixed with cyanoacrylate glue to the thermostated platform below the texture analyzer probe. The probe was then lowered onto the surface of the buccal epithelium with a speed of 0.5 mm/s. A contact force of 0.3 N was applied for 60 s. Afterwards, the materials were separated at a constant speed of 0.1 mm/s. An acquisition rate of 200 points/s and a trigger force of 0.003 N were selected for measurements. Mucoadhesive properties were determined as the strength and the work of mucoadhesion. Cellulose film and commercially available oral gel served as negative and positive controls, respectively. The results are presented as the mean from four independent measurements.

### 2.8. Dissolution Studies

In vitro release of ALA from emulgels was evaluated with a USP dissolution Apparatus II (Agilent 708DS, Agilent Technologies, Cary, NC, USA) through natural cellulose membrane (Cuprophan, molecular weight cut off 10,000 Da, Medicell, London, UK) using an Enhancer cell (diffusion area of 3.80 cm^2^, Agilent Technologies, Cary, NC, USA). About 1.0 g of each emulgel preparation was placed in the drug reservoir on the top of the membrane. The acceptor medium was SSF pH 6.8 (100 mL, 37 ± 0.5 °C) and stirred at 75 rpm. Samples (1 mL) were withdrawn at the predetermined time intervals, filtered through 0.45 µm cellulose acetate filters, diluted with PBS and analyzed using the HPLC method (as described in Section 2.5). Withdrawn samples were replaced with equal volumes of fresh SVF. Reference commercial gel with ALA registered for dermal delivery was applied in control studies. All release experiments were conducted in triplicate.

### 2.9. Ex Vivo Penetration Studies

Penetration experiments were performed using an in-line cell system equipped with thermostated Teflon diffusion chambers (SES GmbH Analysesysteme, Bechenheim, Germany) according to the method described previously by our group [23]. The applied penetration model evaluated the passive diffusion of ALA across porcine buccal tissue. The porcine oral epithelium was placed between the two compartments with the epithelial side facing the donor chamber and conditioned with PBS pH 7.2. Next, the proper amount of each emulgel diluted with SSF, pH 6.2 (which amount referred to 2 mg ALA dose) was carefully applied on the tissue surface. The donor cell remained closed through the studies and the system was maintained at a temperature of 36 ± 1 °C. The acceptor medium consisting of PBS (pH 7.2) and 0.005% sodium azide (as preservative) was then recirculated from the inner side of tissue with a constant rate of 40 mL/h. The drug diffusion area was 0.81 cm^2^. At predetermined time intervals (30, 60, 90, 120 and 180 min), medium samples were withdrawn, filtered and analyzed for ALA content (as described in Section 2.5). The acceptor fluid was refilled to a constant volume of acceptor medium to preserve sink conditions. Reference commercial gel with ALA registered for dermal delivery was applied in control studies.

At the end of the test, emulgels were aspirated from donor compartment to glass flasks and the tissue samples were carefully washed with SSF (pH 6.8) until complete ALA removal from the tissue surface. After 2 h incubation in a thermostated water bath (at 150 rpm, 30 °C), the aspirate was centrifuged (4000 rpm, 15 min), filtered through 0.2 µm cellulose filters and analyzed for ALA content. To assess the amount of ALA retained in the tissue, the porcine buccal tissue was homogenized and incubated in acetonitrile/water (3:7, *v/v*) with 0.1% trifluoroacetic acid for 3 h at 30 °C with gradual shaking at 150 rpm. After the filtration step, the extract was examined for ALA content. Penetration and retention behavior were expressed as the amount of ALA that permeated to the acceptor fluid per tissue unit area. All experiments were performed at least four times.

### 2.10. Safety Profile Using HOE Tissue

#### 2.10.1. Human Cultured Oral Mucosa Model

The reconstituted human oral epithelium tissue HOE (SkinEthicTM HOE, Canton, MA, USA), a three-dimensional, highly differentiated model composed of TR146 cells derived from a squamous cell carcinoma of the buccal mucosa with high resemblance to the human oral mucosa, was applied in the studies. The tissue model was cultured in 0.5 cm^2^ inserts according to the manufacturer’s instructions [24].

#### 2.10.2. Irritation Assay

The test consisted of a topical exposure of emulgels to the HOE model followed by a cell viability assay, which was measured by dehydrogenase conversion of MTT according to the enclosed procedure described in [25]. In brief, inserts with epithelium model were placed onto 300 μL of culture medium (24-well plate) and left overnight (dark, 37 ± 1 °C, 5% CO_2_). Next, inserts with tissue were placed onto 300 μL of fresh maintenance medium (in 24-well plates) for 2 h (dark, 37 ± 1 °C, 5% CO_2_). Then, 30 μL of a 30% dilution of each tested formulation was applied onto tissue and incubated at 37 ± 1 °C, 5% CO_2_. All tested samples were concurrently evaluated on three tissue replicates. Four exposure times of 1, 2, 5 and 18 h were set to verify the predictive performance and reproducibility of the method. Controls were tissues that were exposed to sterile water (negative control, NC) and 5% (*w/w)* sodium dodecyl sulphate (SDS) solution—an anionic surfactant commonly utilized as positive control (PC) for in vitro acute topical irritation studies. After incubation, cultures were rinsed with PBS, transferred to new 24-well plates containing 0.3 mL of MTT solution (0.5 mg/mL) and incubated in the dark at 37 ± 1 °C, 5% CO_2_ for 3 h. Next, each tissue was extracted with 1.5 mL of isopropanol for 2 h, and the extracts were analyzed spectrophotometrically at 570 nm (Dynex Technologies, Chantilly, VA, USA). Each sample was analyzed in triplicate. Percentage of cell viability was calculated as:

% of viability = (absorbance of tested sample/absorbance of negative control) × 100(1)

#### 2.10.3. IL-1β Release Assay

At each incubation time point, post-exposure cell culture media was carefully collected in tubes and stored at −80 °C. IL-1β cytokine level was examined with cytometer BD FACSCanto II (BD Biosciences, San Jose, CA, USA) using a human inflammatory cytokine Cytometric Bead Array (CBA) I Kit (BD Biosciences, San Jose, CA, USA) according to the manufacturer’s instruction. Before analysis, samples were thawed at room temperature and subsequently maintained at room temperature during processing. All samples were centrifuged before transferring onto the assay plate.

#### 2.10.4. Histopathological Analysis

After incubation, one of the triplicate tissue samples from each condition was washed with PBS, fixed in 10% formalin, embedded in paraffin, and sectioned by microtome and submitted to hematoxylin and eosin staining (H&E). Samples were examined under digital microscope (40× magnification, Axiolab 5 microscope with Axiocam camera and ZEN 2.0 software, Zeiss, Sartrouville, France). Each image was studied for morphological changes according to the International Harmonization of Nomenclature and Diagnostic Criteria, INHAND [26].

### 2.11. Statistical Analysis

The results were analyzed using the StatSoft Statistica 13.0 software (StatSoft, Kraków, Poland). The results are presented as arithmetic means ± standard deviations (S.D.). Prior to statistical analysis, normality of the distribution of variables was checked using the Shapiro–Wilk test and the homogeneity of variance was checked using the Levene’s test. The results from mucoadhesive and textural studies were assessed by Kruskal—Wallis tests with a post hoc Dunn’s test. Data from dissolution and penetration studies were analyzed by one-way analysis of variance (ANOVA) with a post hoc Tukey’s test. Measurements were considered significant at *p* < 0.05.

## 3. Results and Discussion

### 3.1. Emulgels’ Characterization

In the present study, the focus was on developing oromucosal ALA-loaded delivery platforms for PDT treatment of precancerous diseases. Despite several research data aimed at evaluating topical ALA-loaded systems in a form of gel composition [27,28,29], to our knowledge this is the first study which has shown the potential of emulgels comprised of natural gums as PDT option for oromucosal application. Prepared formulations displayed off-white color, smooth consistency and the drug content was found uniform and within acceptable limits of 90–110% (Table 3) [30].

**Figure 1:**
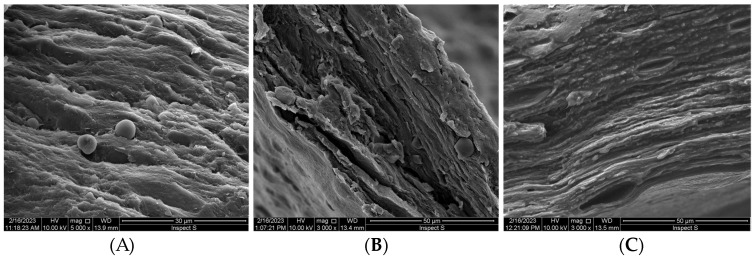
SEM images of F1 (**A**), F2 (**B**), and F3 (**C**) delta-aminolevulinic acid-loaded emulgels (original magnification ×3000 and ×5000).

SEM analysis (Figure 1) showed diversified, layered microstructure of the freeze-dried emulgels with a different level of organization.

The micrographs of formulation F1 (Figure 1A) revealed a laminar network consisting of randomly organized polymer strands with visible bead-like lecithin structures and pores with irregular shape. Formulation F2 appeared more heterogenous, presenting a tangled interconnected pattern of organization (Figure 1B). In turn, emulgel F3 possessed the most compact, organized and laminar network architectures among the tested samples, indicating free miscibility between the GG and XA components (Figure 1C). Based on the SEM micrographs, it can be seen that the presence of XA was responsible for the formation of a more dense, packed network in formulations F1 and F3 (Figure 1A,C). Basically, no drug crystals larger than 1 µm were visible within the samples of all tested emulgels, confirming that ALA was dispersed and uniformly incorporated within a polymer matrix.

**Figure 2:**
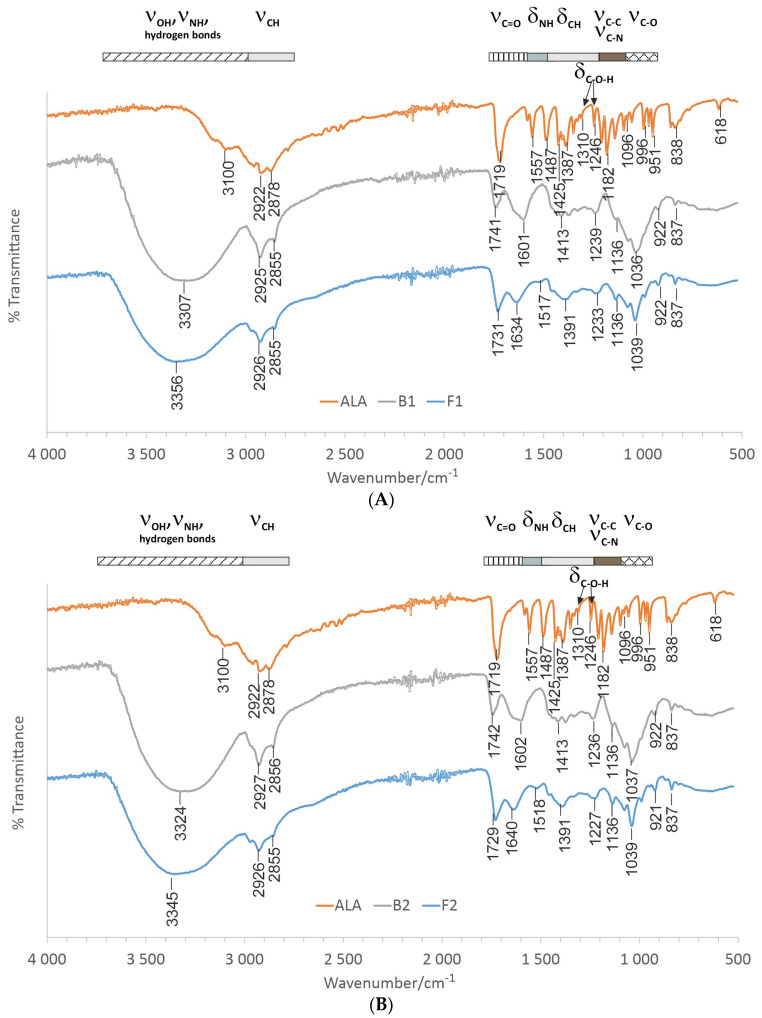

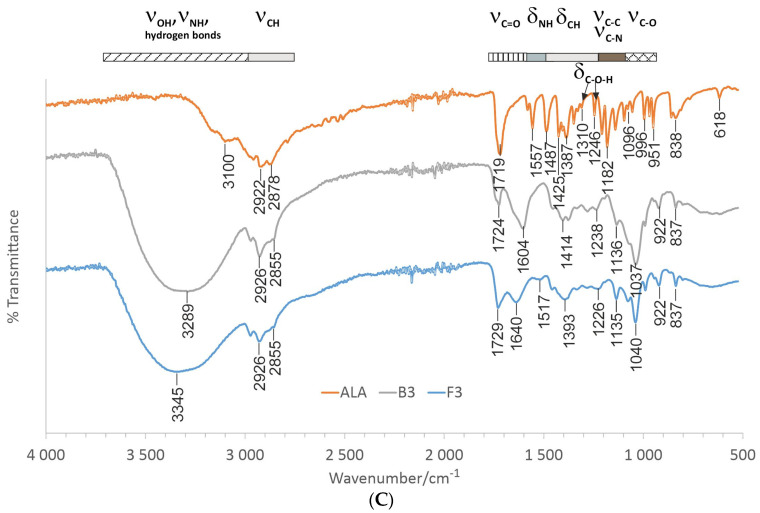
Representative ATR-FTIR of (**A**) tragacanth/xanthan emulgel F1, (**B**) tragacanth emulgel F2 and gellan gum/xanthan emulgel F3 (**C**) when compared to drug-free counterparts B1–B3 and pure delta-aminolevulinic acid (ALA).

Physicochemical analysis was carried out to examine potential interactions between polymers and ALA in the emulgel composition. The ATR-FTIR spectra of formulations F1–F3 with respect to pure drug and placebo counterparts B1–B3 are displayed in Figure 2.

ATR-FTIR spectra of pure ALA showed characteristic peaks for functional groups at wave numbers presented as 1557 cm^−1^ for the N-H bending vibrations of the amino group, 1719 cm^−1^ for the C=O stretching vibrations of carbonyl and carboxyl groups, 1310 cm^−1^, 1245 cm^−1^ for the C-O-H bending vibrations and 1096 cm^−1^ for C-O stretching vibrations of the carboxylic acid [31]. The spectra of ALA-loaded emulgels F1–F3 confirmed the presence of surface-adsorbed drug particles. Higher intensity in the ALA absorption band representative of the C=O bond at 1730 cm^−1^ was visible in spectra of F1–F3. In general, the spectral peaks of investigated formulations were found to be comparable to those obtained for the pure drug, confirming that ALA is compatible with emulgel compositions.

### 3.2. Texture Analysis

**Figure 3:**
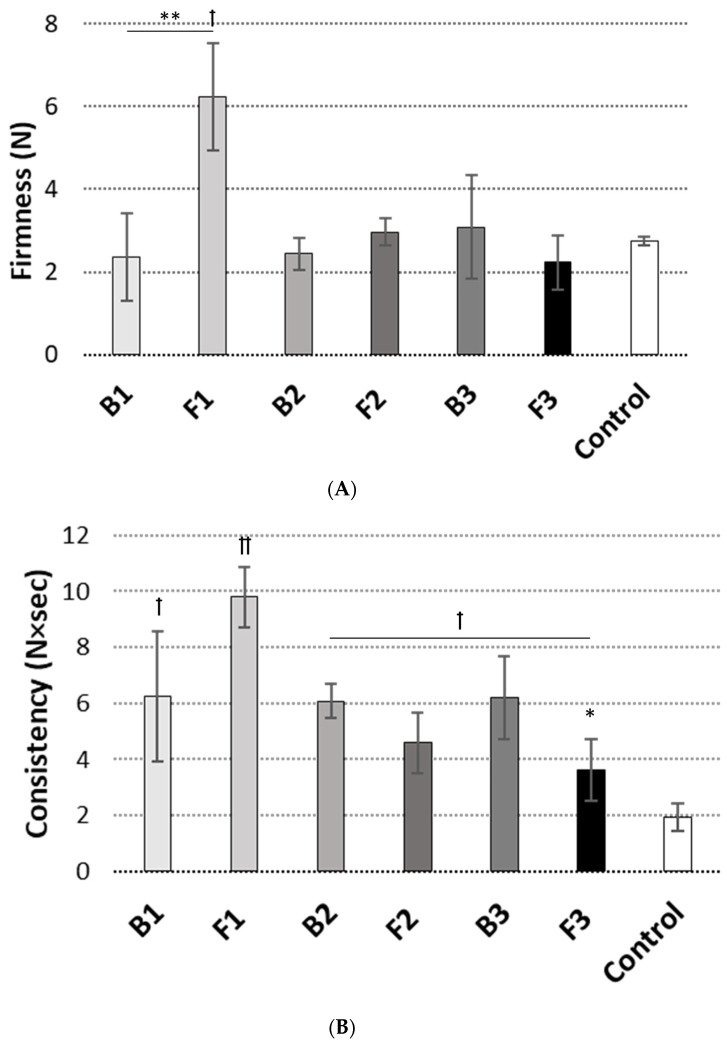

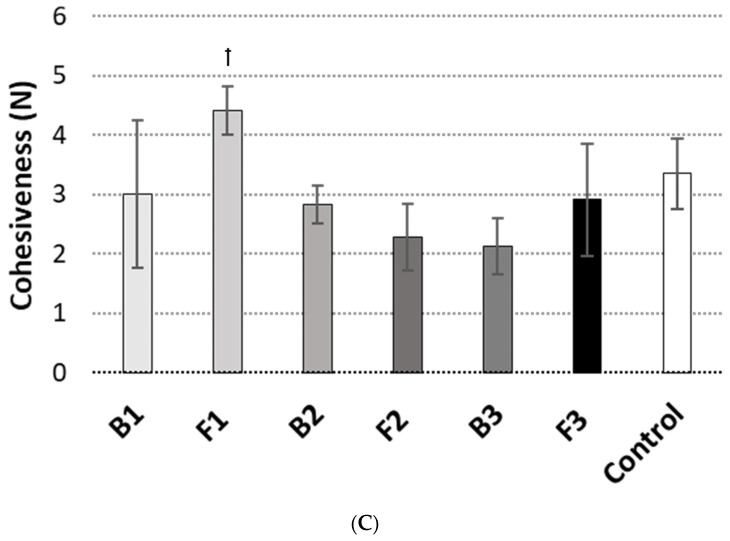
Textural behavior: (**A**) hardness, (**B**) consistency and (**C**) cohesiveness of ALA-loaded emulgels F1–F3 compared to drug-free preparations B1–B3 and control (reference oromucosal gel) (*n* = 3; mean ± S.D.). * and ** represent differences between drug-free and ALA-loaded formulations with *p* ≤ 0.05 and with *p* ≤ 0.01, respectively; † and †† symbolize differences with *p* ≤ 0.05 and *p* ≤ 0.01, respectively as compared to control.

Figure 3 presents the textural characteristics, namely hardness, consistency and cohesiveness, of the designed ALA-loaded emulgels compared to drug-free formulations and reference oromucosal gel. In our studies, hardness (or firmness) refers to the maximum force that emulgel can handle, consistency reflects its ability to deform under stress and spreading on the mucosal surface, whereas cohesiveness (connected with the breaking of cohesive bonds) describes the sample’s recovery after initial stress.

As Figure 3A,C display, all tested drug-free preparations were relatively firm, presenting comparable values of hardness and cohesiveness to those attained with the reference oromucosal gel product (Figure 3A,C). Approximately twofold greater values of consistency were obtained for these formulations. This pointed to their more compact structure and suggested that more work may be required to spread them over the mucosal surface (Figure 3B). Basically, the presence of XA appeared not to have any real effect on the mechanical properties as no real differences were noticed between formulations B1 and B2. The incorporation of an active agent affected textural behavior of emulgels F1 and F3. Formulation F1 displayed about 80 and 30% increases in hardness and cohesiveness values, respectively, in comparison to drug-free base B1 (Figure 3A,C). In turn, comparable values of textural properties were found between B2 and F2. The greater cohesive interactions present in the F1 sample could have resulted from the polyelectrolyte complex formation between ALA amino groups (dominating at pH < 5.0) and large density of carboxylic moieties present in XA and TG [14]. These electrostatic interactions may be considered as factors supporting retention capability [32]. In contrast, about 30% loss of hardness and consistency was noted for emulgel F3 comprising GG and XA. By acidifying the environment, ALA could have made GG/XA less negatively charged and stopped PEC formation, which in turn decreased mechanical properties.

### 3.3. Mucoadhesive Behavior

**Figure 4:**
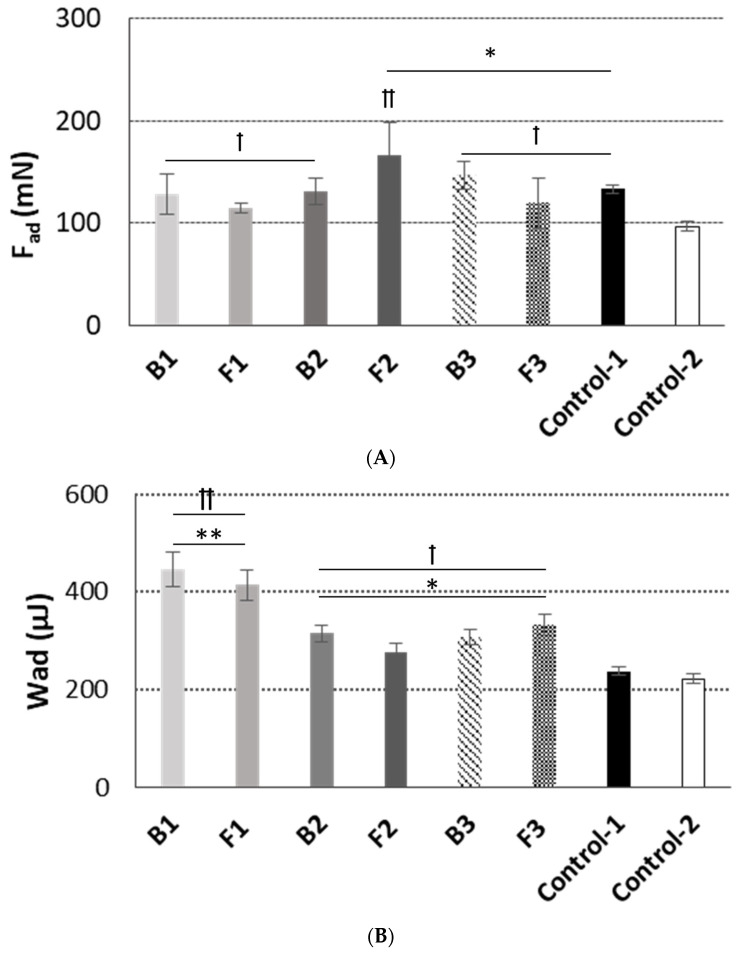
Mucoadhesive behavior: (**A**) maximum force of detachment, (**B**) work of adhesion of ALA-loaded emulgels F1–F3 as compared to drug-free formulations (B1–B3) and controls (Control-1—reference commercial oromucosal gel; Control-2—reference topical gel with ALA) (mean ± S.D.; *n* = 4). * and ** represent differences between emulgel formulations and Control-1 with *p* < 0.05 and with *p* < 0.01, respectively; † and †† symbolize differences with *p* < 0.05 and *p* < 0.01, respectively as compared to Control-2.

Next, ex vivo mucoadhesive behavior of the designed emulgels in contact with excised porcine buccal mucosa was assessed by tensometric measurements. The mucoadhesive tissue model was selected based on the anatomical and structural resemblance to human oral epithelium [33]. In our studies, the maximum detachment force parameter reflected the mechanical stress (including tongue movements) responsible for interrupting contact between the formulation and oromucosal tissue. In turn, the work of mucoadhesion imitated the capability of retention on the mucosal site after application. Data attained for formulations F1–F3 are presented in Figure 4 and compared with those obtained for placebo formulations (B1–B3), a reference commercial oromucosal gel (Control-1) and a reference commercial topical gel with ALA (Control-2).

The examined samples responded differently upon contact with mucosal tissue. Basically, the combination of TG and XA was found to strengthen the overall ability to interact with mucosal tissue (Figure 4B). The presence of XA improved the mucoadhesive capacity of TG/XA-based formulation B1, which resulted in an increase in mucoadhesion of about 60% when compared to the TG-based formulation B2. In turn, the combination of GG and XA in formulation B3 exhibited approximately 30% lower values of mucoadhesiveness than those obtained for formulation B1 (Figure 4B), showing its weaker ability to interact with tissue.

It should be noted that all designed emulgels displayed greater overall ability to interact with mucosal tissue when compared to Control-2, a commercial topical gel containing ALA. Importantly, the designed formulations were characterized by higher values of adhesion than those obtained for the reference gel for oromucosal injuries Control-1. In particular, TG/XA-based emulgel B1 (and its drug-loaded counterpart F1) was distinguished from the other preparations as it exhibited a twofold enhancement in retention on the mucosal tissue as compared to Control-1 (Figure 4B). Notably, the interaction between emulgel and porcine cheek was not altered by the presence of ALA. That indicated the applied polymer gums were insensitive to the presence of an acidic drug substance. Surprisingly, formulation F2 displayed even higher values of detachment force (vs. base B2), which suggests it may be relatively more resilient to sharp tension, e.g., tongue movements (Figure 4A). Overall, formulation F1, with greater adhesion, and formulation F2, with the highest detachment force, showed the most favorable retention on oromucosal tissue.

### 3.4. Dissolution Studies

**Figure 5:**
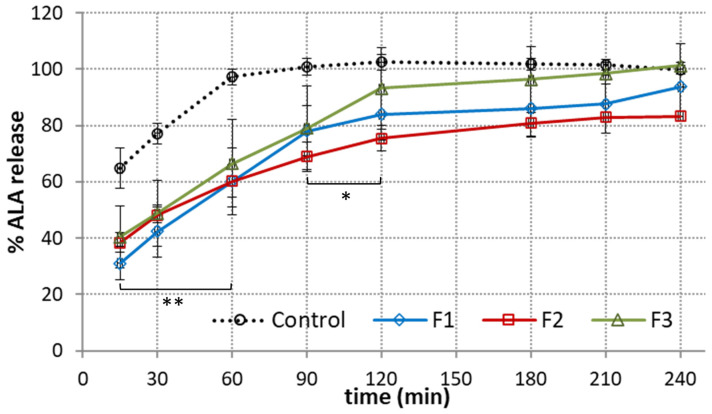
In vitro ALA dissolution profile from emulgels F1–F3 compared to reference commercial gel with ALA (Control) in simulated saliva fluid, pH 6.8 (mean ± S.D.; *n* = 3); * and ** represent differences in release behavior between emulgels and control with *p* < 0.05 and with *p* < 0.01, respectively.

Next, the effect of polymer composition on the ALA release profile from the emulgels was investigated. A comparison between the cumulative ALA release from emulgels F1–F3 in SFF pH 6.8 is presented in Figure 5.

In terms of oral or buccal delivery, it is important to assure a relatively fast and steady drug release rate before the dosage form will be cleared from the application site [12]. From Figure 5, it can be seen that ALA was gradually released from the tested preparations. A gradual dissolution was obtained for tested emulgels with about 40–50% of drug present in the acceptor medium within the first 30 min of the test. In turn, more rapid onset was obtained in control studies, with a burst effect of about 70% attained within the first 15 min. A complete ALA release from the reference product was reached after 60 min. The observed immediate dissolution of the reference product may lead to fast washing out of drug from the application site. In our studies, the time required for 80% of ALA release from the designed emulgels was moderately prolonged up to 90–120 min. Formulation F2 demonstrated the slowest release rate among the tested formulations. This observation may be associated with a complex network architecture and tangled interconnected pattern of organization (Figure 1B). In terms of oromucosal carriers, a moderate drug release profile together with mucoadhesive properties appear desirable to assure drug therapeutic levels at the absorption site. Interestingly, the blend of XA and TG in formulation F1 gave a more steady drug release profile at the initial stage of the test, with the amount of drug below 30% after the initial 15 min of studies.

### 3.5. Penetration Studies

**Figure 6:**
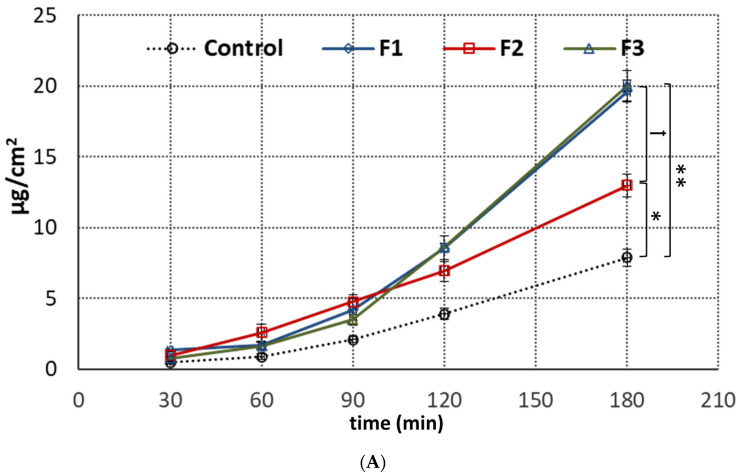

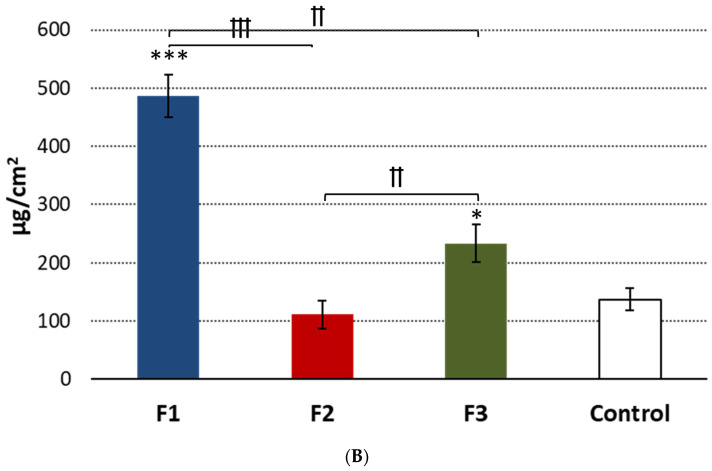
Ex vivo penetration (expressed as the amount of ALA in acceptor fluid per tissue surface area) (**A**) and retention (**B**) from emulgels F1–F3 and control (reference commercial gel with ALA) through porcine oromucosal epithelium (mean ± S.D; *n* = 5). *, ** and *** represent differences between control and emulgels with *p* < 0.05, *p* < 0.01 and with *p* < 0.001, respectively. †, †† and ††† symbolize differences between emulgels with *p* < 0.05, *p* < 0.01 and *p* < 0.001, respectively.

Penetration studies are an important element in evaluating the quality of topical formulations that enable prediction of in vivo topical absorption [34,35]. Despite a number of research studies investigating the penetration behavior of ALA through the skin barrier, to our best knowledge hardly any data exist on its penetration and retention profile across oromucosal epithelium. Therefore, in the present studies, the passive diffusion of ALA from designed emulgels across an excised porcine oromucosal epithelium was evaluated. The particular focus was on elaborating the effect of emulgel composition and the type of polymer used in emulgel preparation on ALA penetration behavior. Figure 6A shows the permeated fraction of ALA from emulgels F1–F3 and reference gel with ALA over time across porcine oromucosal epithelium. The amount of ALA recovered from the donor compartment varied between 93 and 98% and fulfilled the criterion of acceptation of overall recovery (90–100%) [36].

Basically, formulations F1 and F3 containing XA were found to increase ALA permeability across oromucosal tissue, which was particularly visible within the second part of the test. In turn, TG-based emulgel F2 showed a slower rate of drug diffusion (with an approximately 30% lower drug concentration in acceptor medium after 2 h and 3 h of the studies when compared to F1 and F3 (*p* < 0.05). It is worth noting that all designed emulgels exhibited significantly greater permeability than that observed in the control test with reference topical gel with ALA (Figure 6A). This may be due to lecithin and castor oil being present in the emulgel composition (Table 2), which are excipients with known penetration-enhancing properties favoring ALA diffusion across the oromucosal membrane [18]. However, the presence of XA appeared to have a beneficial effect on ALA absorption as well, since formulations F1 and F3 displayed a profoundly higher ALA penetration rate (*p* < 0.01). The above observations are in accordance with the studies performed by the Shiledar group, which revealed that designed buccal XA-patches increased zolmitriptan permeability across sheep buccal mucosa [37]. Interestingly, emulgels F1 and F3 also showed greater accumulation in tissue as compared to formulation F2 and the reference gel with ALA (Figure 6B). In particular, the combination of XA and TG in emulgel F1 acted as an absorption enhancer and increased ALA retention in mucosal tissue fourfold with regard to TG-based formulation F2. Numerous research papers have pointed out that ALA is absorbed into cells by two different mechanisms, passive diffusion and active transport [38,39]. In contrast, Gederaas et al. reported that ALA crosses the membranes through active transport while passive diffusion is negligible [40]. The presented studies revealed that ALA is capable of penetrating across oromucosal epithelium by passive transport. Regardless of the mode of drug transport, the penetration of ALA through intact biological barriers (including skin) is considered low, making it difficult to achieve the desired therapeutic benefits. Different strategies were proposed to improve these penetration issues, including modifying ALA structural properties, or altering the skin state by physical or chemical enhancement methods [7,41]. Here, we discovered that dosage form composition plays a substantial role in enhancing the rate of ALA diffusion across the mucosal epithelium. Importantly, by introducing XA into emulgels’ structure, the absorption and accumulation rates of ALA were modulated, which in turn may improve its therapeutic efficacy at the application site.

### 3.6. Safety Profile

#### 3.6.1. Irritation Studies

The intimate contact between the mucoadhesive drug delivery system and the mucosal tissue requires preparations to be nontoxic in order not to cause any irritation or injury. Therefore, the important aspect of this study was to evaluate the safety profile of the designed ALA-loaded emulgels and corresponding drug-free preparations on the HOE tissue model according to EVCAM requirements [42]. In our studies, four exposure times were chosen: 1, 2, 5 and 18 h as the last time point imitating repeated dosing upon a treatment scheme.

**Figure 7:**
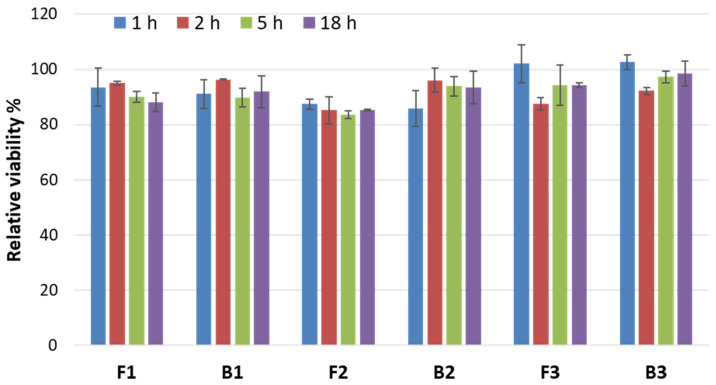
MTT viability of human oral epithelium model after 1, 2, 5 and 18 h incubation with designed drug-free emulgels (B1–B3) and formulations containing ALA (F1–F3) expressed as percentage of negative control (NC, water treated tissue); viability of positive control assessed after 1 and 2 h incubation was 48 ± 0.5% and 15 ± 0.9%, respectively (mean ± S.D.; *n* = 3).

Initially, emulgels were evaluated for their irritation potential in contact with the HOE model using MTT assay, and the results are presented in Figure 7. Basically, relative to the HOE cell survival exposed to water (NC, set at 100%), the mean percentage viability for emulgels was above 80% at all tested time points. A slight decrease in metabolic activity (below 86%) was observed only for formulation F2 upon 2, 5 and 18 h exposure. The presence of ALA in emulgels F1–F3 had no real impact on the tissue’s metabolic activity when compared to drug-free formulations. Some higher survival rate was noted for samples F3 and B3, except for at time point 2 h, at which formulation F1 and B1 displayed greater viability values.

#### 3.6.2. IL-1β Release Assay

**Figure 8:**
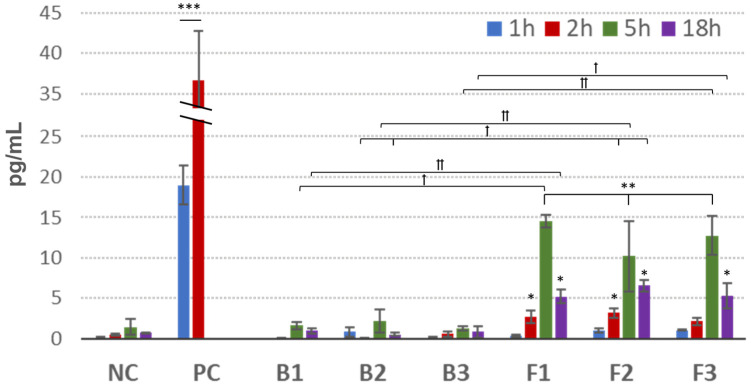
Concentration of interleukin IL-1β in the culture media after exposure of 3D human oral mucosa model to ALA-loaded emulgels (F1–F3) and corresponding drug-free formulations (B1–B3) compared to negative control (NC, water treated tissue) and positive control (PC, tissue treated with 5% sodium dodecyl sulphate) (mean ± S.D.; *n* = 3) *, ** and *** represent differences in comparison to NC with *p* ≤ 0.05, *p* ≤ 0.01 and with *p* ≤ 0.001, respectively; † and †† symbolize differences between drug-free and corresponding ALA-loaded formulations with *p* ≤ 0.05 and *p* ≤ 0.01, respectively.

The IL-1β release into culture media was measured alongside MTT assay as the second point for the prediction of samples’ irritancy. The proinflammatory effects were analyzed by cytometric assay and are presented in Figure 8.

The IL-1 family (a group of cytokines comprised of IL-1α and IL-1β) is identified as key interleukins released from keratinocytes in response to inflammatory agents, infections and microbial endotoxins [43]. IL-1 is constitutively expressed in a variety of epithelial cells in order to maintain the epithelial barrier and defend cells against injury [44]. In particular, IL-1β has been identified as a potent inducer of inflammation of periodontal tissue and linked to periodontal diseases and gingivitis [45].

As shown in Figure 8, the HOE model responded differently to tested samples in terms of IL-1β release. Compared with negative control, no real impact of drug-free formulations B1–B3 on the level of IL-1β was observed. In turn, ALA-loaded emulgels exhibited time-dependent increase in the quantity of IL-1β. This may be related to a non-specific cytotoxic effect exerted by ALA related to acidic behavior and the decreasing pH of culture medium. That in turn may have initiated the HOE model response. A moderate rise in the concentration of IL-1β (up to 10.2 and 14.5 pg/mL for samples F2 and F1, respectively) (*p* < 0.05) was principally visible after 5 h incubation. Interestingly, upon 18 h exposure, a 2- and 3-fold drop in cytokine concentration was noted compared to that observed after 5 h incubation. This may be attributed to a short half-life of IL-1β (approximately 4 h) in the culture medium [46,47] or associated with the HOE model aging. According to Pilkington et al., both IL-1β gene expression and IL-1β mRNA levels remained lower in aged skin samples ex vivo compared with a young skin model [48].

No statistically significant differences between tested emulgels F1–F3 or between drug-free bases B1–B3 on the IL-1β release were noted. Noteworthily, all tested formulations F1–F3 exerted a moderately low effect on IL-1β release when compare to PC, with an approximately 35-fold greater value of IL-1β than NC after 2 h (Figure 8). Additionally, no rapid onset was observed in IL-1β concentration after 1 h incubation with emulgels F1–F3. In contrast, PC caused immediate release of IL-1β with an approximately 20-fold increase over NC after 1 h incubation.

According to the EU and GHS classification (R38/Category 2 or no label), an irritant potential is predicted for a preparation when the mean relative tissue viability is reduced below 50% of the mean viability assessed for negative control and IL-1 release reaches values ≥ 50 pg/mL [42]. All samples showed negligible reduction in cell viability and moderately low release of IL-1β, confirming their non-irritancy and compatibility with the oral epithelium model.

#### 3.6.3. Histological Analysis

**Figure 9:**
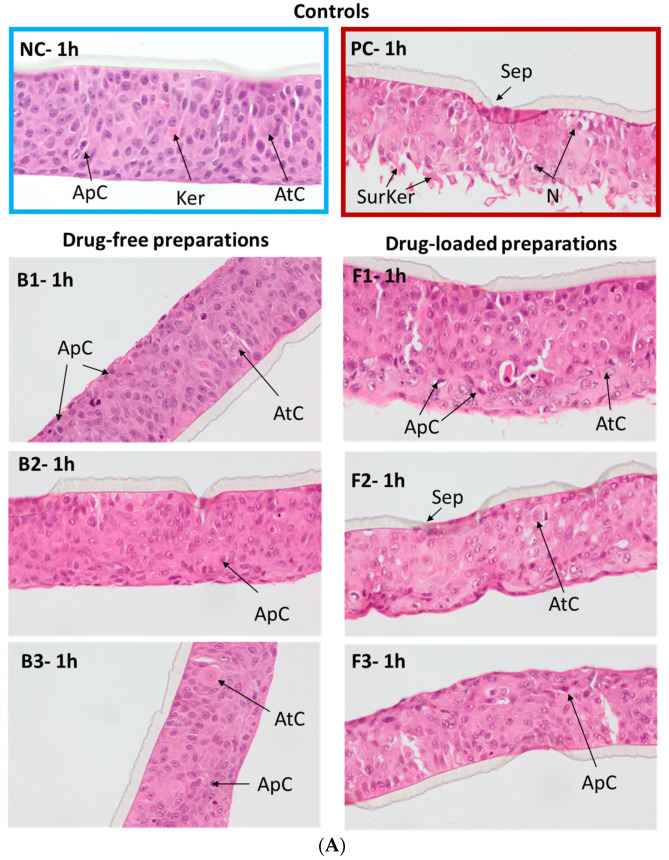

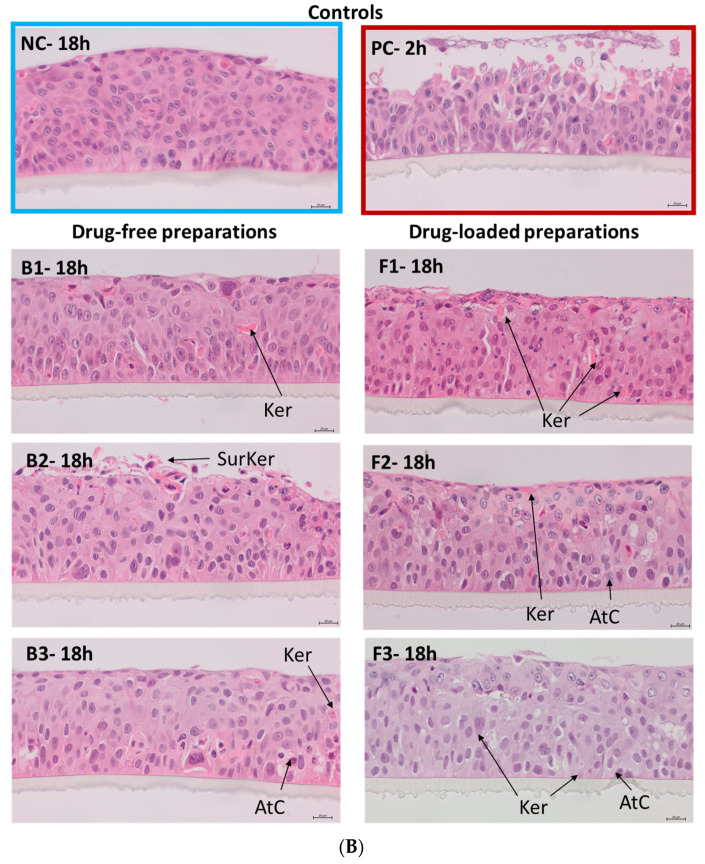
H&E staining of the representative histopathological sections of the human oral epithelium model after (**A**) 1 h, and (**B**) 18 h incubation with ALA-loaded emulgels (F1–F3) and corresponding drug-free formulations (B1–B3) compared to negative control (NC, water treated tissue) and positive control (PC, SDS treated tissue). ApC—apoptotic cell; AtC—atypic cell; Ker—keratinization; SurKer—surface keratinization; N—necrotic cell; Sep—separation from connective tissue.

To further examine the emulgels’ safety profile, histological analysis was carried out on the HOE tissue model specimens after H&E staining (Figure 9). At each time point, tissue samples were investigated for morphological abnormalities, particularly for the signs of atypia, keratinization, apoptosis, necrosis and the level of integrity of the connective tissue layer. From the above, the most cytotoxic irreversible alterations were necrosis characterized by cytoplasm swelling and distortion of organelles, and keratinization with loss of membrane integrity.

Figure 9A shows the histological analysis of the tissue model upon 1 h incubation with emulgels and controls. The tested emulgels caused no significant damage to the oral mucosa model. The histopathological evaluation of tissue samples exposed to designed F1–F3 formulations and corresponding B1–B3 placebo emulgels was comparable to that of the control NC tissue samples treated with water. As can be seen in Figure 9, the stratum corneum of examined tissue samples was intact and composed of viable, normal morphology keratinocytes with few atypic cells. The single apoptotic cells present in all tissues, including the NC-treated group, indicated normal cell turnover during tissue culture.

In contrast, the positive control SDS presented visible signs of keratinization, loss of integrity and cells separation from the connective tissue scaffold. As presented in Figure S1A, damage deepened over time and marked epithelial necrosis occurred after 2 h incubation in PC-treated tissue. In addition, fragmentation and damage to the connective tissue scaffold was observed with a loss of the normally distinct boundary between the scaffold and the epithelium. Histopathologic evaluation upon 2 and 5 h did not reveal obvious morphological or structural alterations in the emulgel-exposed HOE tissues. Figure S1B shows that the cellular membrane remained intact with no damage to the epithelial layer and no signs typical of tissue irritation in all tested samples.

Some morphological changes, however, occurred after 18 h exposure, particularly in tissues incubated with ALA-loaded emulgels (Figure 9B). Analyses of the H&E-stained tissue sections exposed to F1–F3 formulations displayed marked atypia and profound signs of keratinization indicating gradual degeneration of the cells (Figure 9B). Basically, the level of abnormalities appeared to be unspecific among ALA-loaded emulgels. Slightly higher cytotoxic effect was observed for formulation F2, where a reduction in epithelium thickness was noted. In turn, the placebo group had a low (formulation B1) to moderate influence (formulations B2 and B3) on the cellular morphology and the level of keratinization of the HOE model.

The results of the histology and cytotoxicity assay demonstrated that placebo emulgels B1–B3 did not influence cell viability and had no observable adverse effects on the oral mucosal model within an 18 h incubation period. Minor signs of HOE irritation after long-term incubation with ALA-loaded formulations was noted when compared to placebo and NC-treated tissues. These observations are in line with data from the IL-1 β assay, suggesting the presence of ALA may be responsible for epithelial dysfunctions but only upon repeated dosing and long-term treatment. Further in vivo studies involving human subjects will carefully examine the safety profile and therapeutic efficacy of designed ALA-loaded emulgels on premalignant lesions of the soft oral tissues.

## 4. Conclusions

The presented findings show the potential of designed emulgels comprised of TG, XA or GG as biocompatible oromucosal platforms for ALA. Mucoadhesive measurements displayed greater ability to interact with porcine mucosa of emulgels comprised of TG/XA when compared to a commercial topical product with ALA. Importantly, we discovered that ALA is capable of penetrating across the oromucosal epithelium by passive diffusion and the type of polymer gum in the emulgel composition affected photosensitizer permeability behavior across the mucosal barrier. All samples showed negligible reduction in cell viability and low release of IL-1β, confirming their non-irritancy and compatibility with the oral epithelium model. Histological analysis demonstrated no significant effect on the oral mucosal model upon 5 h incubation. The minor signs of epithelial dysfunctions after 18 h incubation may suggest the negative impact of the photosensitizer itself on tissue upon repeated dosing or long-term application. Overall, emulgel comprised of TG/XA with favorable mucoadhesive properties, steady drug release profile and improved ALA accumulation in epithelial tissue was found the most promising carrier, holding promise as an oromucosal ALA platform for PDT strategy. Further in vivo studies will examine its therapeutic efficacy on premalignant lesions of the soft oral tissues.

## 5. Patents

Szymańska et al. (2023), Patent Application number P.443813 (PL).

## Figures and Tables

**Table 1:** List of substances used in the studies.

Name of Chemical	Company
Acetonitryl (HLPLC grade)	Avantor Performance (Gliwice, Poland)
Castor oil (pharmaceutical grade)	Coel (Kraków, Poland)
Delta-aminolevulinic acid hydrochloride (purity ≥ 99%, serial number S005/syntal/19022020)	Syntal Chemicals Sp. Z o.o. (Gliwice, Poland)
Delta-aminolevulinic acid hydrochloride (internal standard)	Sigma Aldrich (Steinheim, Germany)
Disodium dihydrogen ethylenediaminetetraacetate	ChemPur (Piekary Śląskie, Poland)
Fluorescamine	Sigma Aldrich (Steinheim, Germany)
Phosphate buffer saline (PBS)	BTL (Łódź, Poland)
Propylene glycol	Avantor Performance (Gliwice, Poland)
Sodium benzoate	ChemPur (Piekary Śląskie, Poland)
Sodium chloride	Polpharma (Starogard Gdański, Poland)
Sodium dodecyl sulfate (SDS)	Avantor Performance (Gliwice, Poland)
Soybean phosphatidylcholine (Phospholipon 90)	Lipoid (Kőln, Germany)
Trichloroacetic acid (HPLC grade)	Avantor Performance (Gliwice, Poland)

**Table 2:** Composition of ALA-loaded emulgels F1–F3 and corresponding drug-free preparations B1–B3.

Compound	Concentration (%, *w/w*)					
	B1	B2	B3	F1	F2	F3
5-aminolevulinic acid	-	-	-	5.0	5.0	5.0
Tragacanth gum	5.0	5.0	-	5.0	5.0	-
Xanthan gum	1.0	-	2.0	1.0	-	2.0
Gellan gum	-	-	0.7	-	-	0.7
Lecithin	0.5	0.5	0.5	0.5	0.5	0.5
Castor oil	2.0	2.0	2.0	2.0	2.0	2.0
Disodium dihydrogen ethylenediaminetetraacetate	0.1	0.1	0.1	0.1	0.1	0.1
Sodium benzoate	0.1	0.1	0.1	0.1	0.1	0.1
Propylene glycol	5.0	5.0	5.0	5.0	5.0	5.0
Purified water	up to 100.0	up to 100.0	up to 100.0	up to 100.0	up to 100.0	up to 100.0

**Table 3:** Drug content and pH values of emulgels F1–F3 after preparation and upon 3-month storage at 4 °C (*n* = 3; mean ± S.D.).

	Emulgel F1	Emulgel F2	Emulgel F3
	After preparation		
Drug content (%)	94.2 ± 2.2	95.3 ± 1.8	91.6 ± 1.5
pH	3.75 ± 0.02	3.73 ± 0.03	3.91 ± 0.01
	Upon 3-month storage at 4 °C		
Drug content (%)	92.4 ± 1.3	94.2 ± 3.2	92.0 ± 1.7
pH	3.78 ± 0.04	3.77 ± 0.02	3.86 ± 0.03

## Data Availability

The data presented in this study are available on request from the corresponding author.

## Supplementary Materials

The following supporting information can be downloaded at: https://www.mdpi.com/article/10.3390/pharmaceutics15102512/s1, Figure S1: H&E staining of the representative histopathological sections of the human oral epithelium model after (**A**) 2 h, (**B**) 5 h incubation with ALA-loaded emulgels (F1–F3) and corresponding drug-free formulations (B1–B3) compared to negative control (NC, water treated tissue) and positive control (PC, SDS treated tissue).
