# Supplementary material for: Evaluation of Oromucosal Natural Gum-Based Emulgels as Novel Strategy for Photodynamic Therapy of Oral Premalignant Lesions
Source: Pharmaceutics. 2023 Oct 23;15(10):2512. doi: 10.3390/pharmaceutics15102512 (PMC10610218; doi:10.3390/pharmaceutics15102512)
Supplement: Supplementary file 1 [file pharmaceutics-15-02512-s001.zip › pharmaceutics-2657844-supplementary.pdf]

Supplementary Material

Article

Evaluation of Oromucosal Natural Gum-Based Emulgels as Novel Strategy for Photodynamic Therapy of Oral Premalignant Lesions

Emilia Szymańska [1,*], Joanna Potaś [1], Marcin Baranowski [2], Robert Czarnomysy [3], Magdalena Ewa Sulewska [4],
Anna Basa [5], Małgorzata Pietruska [4], Krzysztof Bielawski [3] and Katarzyna Winnicka [1]

1 Department of Pharmaceutical Technology, Medical University of Białystok, Mickiewicza 2c, 15-222 Białystok, Poland; joanna.potas@umb.edu.pl (J.P.); katarzyna.winnicka@umb.edu.pl (K.W.)
2 Department of Physiology, Medical University of Bialystok, Mickiewicza 2c, 15-222 Białystok, Poland; marcin.baranowski@umb.edu.pl
3 Department of Synthesis and Technology of Drugs, Medical University of Białystok, Jana Kilińskiego 1, 15-089 Bialystok, Poland; robert.czarnomysy@umb.edu.pl (R.C.); krzysztof.bielawski@umb.edu.pl (K.B.)
4 Department of Periodontal and Oral Mucosa Diseases, Medical University of Białystok, ul. Waszyngtona 13, 15-269 Białystok, Poland; magdalena.sulewska@umb.edu.pl (M.E.S.); mpietruska@wp.pl (M.P.)
5 Faculty of Chemistry, University of Bialystok, Ciołkowskiego 1K, 15-245 Białystok, Poland; abasa@uwb.edu.pl
* Correspondence: emilia.szymanska@umb.edu.pl; Tel.: +48-8574-856-16

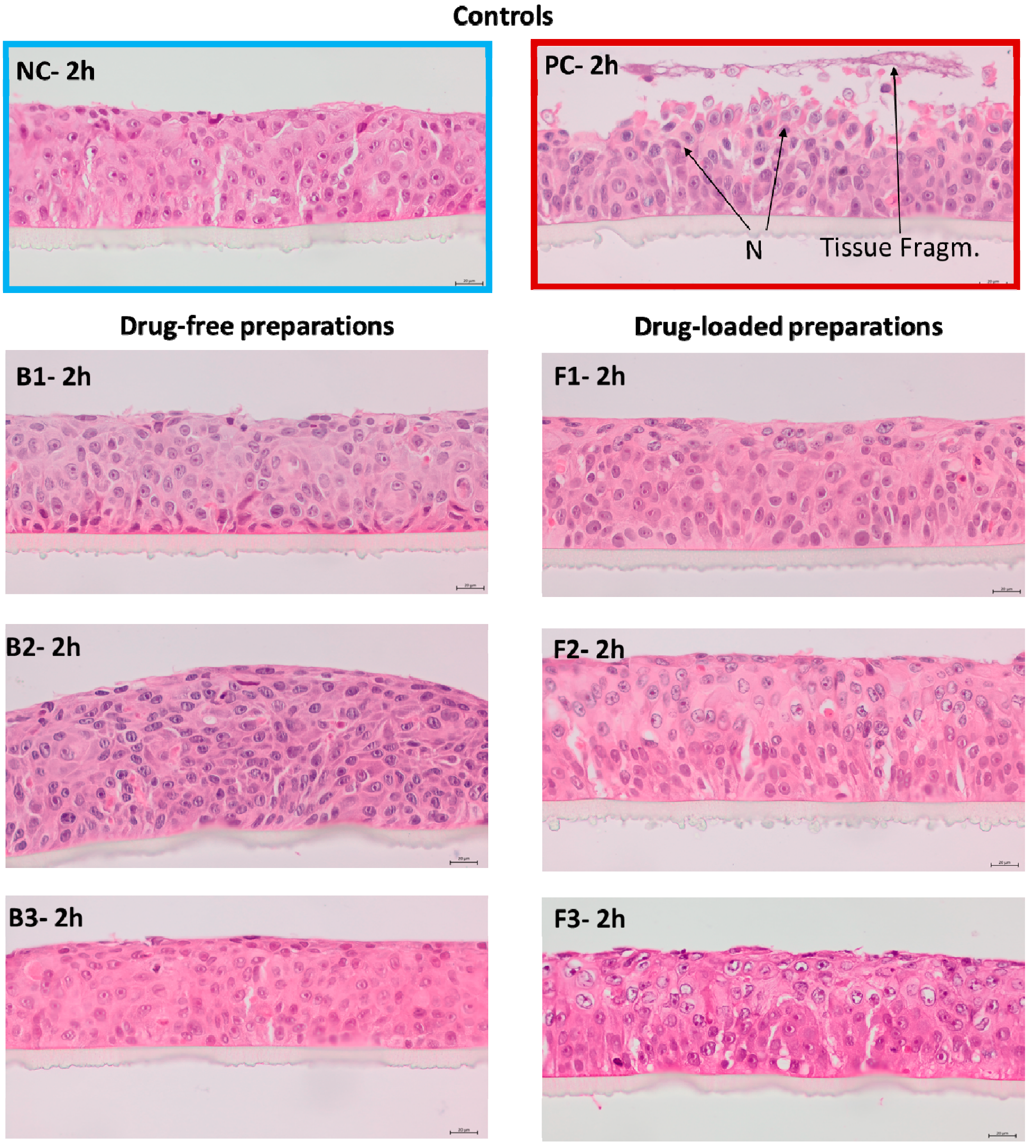
Controls
NC- 2h
PC- 2h
N
Tissue Fragm.
Drug-free preparations
Drug-loaded preparations
B1- 2h
F1- 2h
B2- 2h
F2- 2h
B3- 2h
F3- 2h

(A)

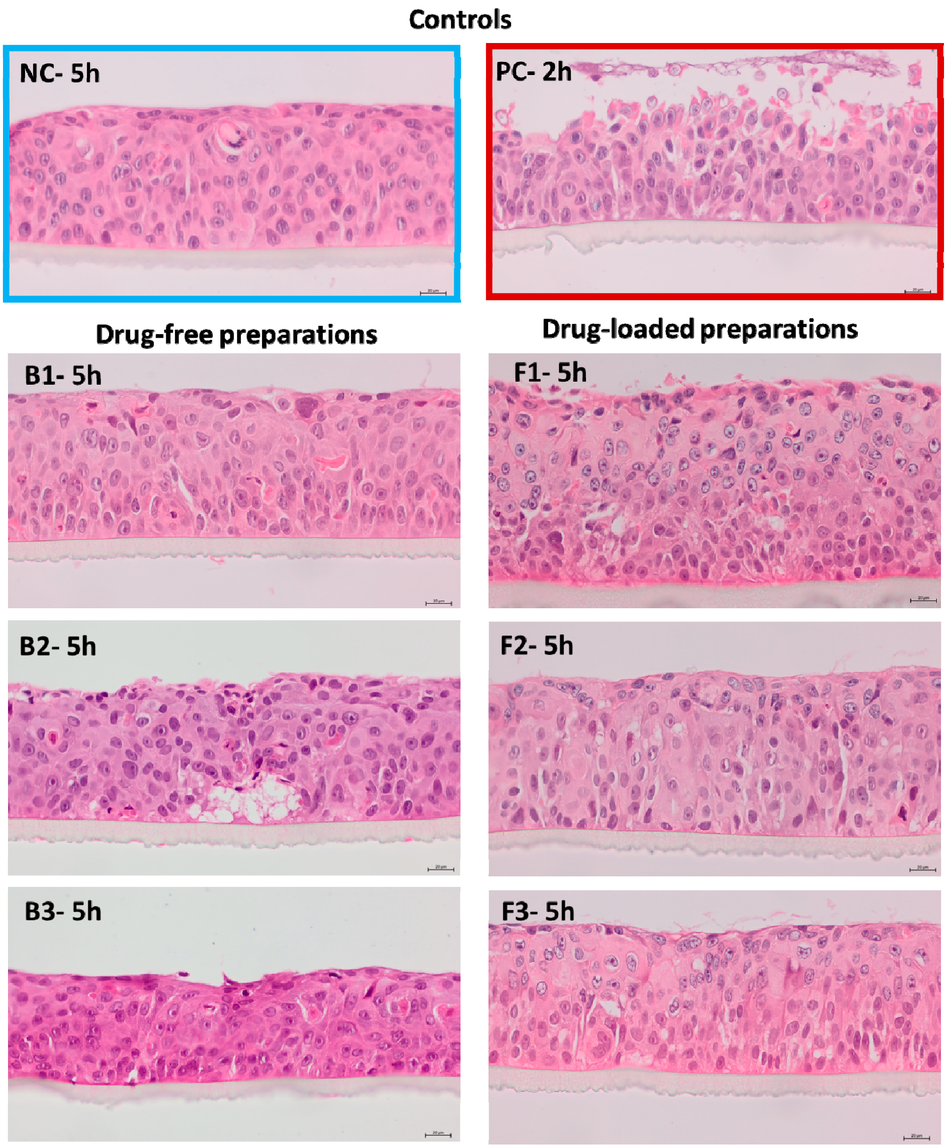

(B)

Figure S1. H&E staining of the representative histopathological sections of the human oral epithelium model after: (**A**) 2h, (**B**) 5h incubation with ALA-loaded emulgels (F1-F3) and corresponded drug-free formulations (B1-B3) compared to negative control (NC, water treated tissue) and positive control (PC, SDS treated tissue). Tissue Fragm. – tissue fragmentation; N – necrotic cell.
